# Effects and Mechanism of Baicalin on Apoptosis of Cervical Cancer HeLa Cells *In-vitro*

**Published:** 2015

**Authors:** Yong Peng, Zhan-zhao Fu, Cong-Shan Guo, Yan-Xia Zhang, Ya Di, Bin Jiang, Qing-Wang Li

**Affiliations:** a Department of Biomedical Engineering, College of Electrical Engineering, Yanshan University, Qinhuangdao, 066004, China.; b Department of Biological Engineering, College of Environment, Chemical Engineering, Yanshan University, Qinhuangdao, 066004, China.; c Department of Animal Science, Northwest Agriculture and Forestry University, Yangling, 712100, China.; d The First Hospital of Qinhuangdao Town, Qinhuangdao, 066100, China.

**Keywords:** Baicalin, HeLa cells, Cell apoptosis, Mitochondrial pathway, Death receptor pathway, Caspase-3

## Abstract

The objective of this study was to observe the apoptosis-inducing effect and mechanism of baicalin on human cervical cancer HeLa cells. The inhibitory effect of baicalin on the growth of HeLa cells was measured by MTT assay, and cell proliferation and migration was analyzed by cell scratch assay. Morphological changes of apoptotic cells were viewed by the light microscope and electron microscope, and cell growth arrest was confirmed by flow cytometry. Moreover, Western blot was used for investigating the expression of apoptosis related proteins; spectrophotometry was used to examine Caspase-3 activation. Our results showed that baicalin could inhibit the proliferation of HeLa Cells via induction of apoptosis in a time and dose-dependent manner (*P*<0.01). Apoptotic signaling induced by baicalin was characterized by up-regulating Bax, Fas, FasL and Caspase-8 protein expression, and down-regulating of Bcl-2 protein expression. These results indicated that baicalin-induced apoptosis involved activation Caspase-3 in HeLa cells through the intracellular mitochondrial pathway and the surface death receptor pathway.

## Introduction

Baicalin, a flavonoid which is extracted from a traditional Chinese herb *Scutellaria baicalensis* Georgi and possesses many significant biological activities, such as anti-oxidant, anti-inflammatory, anti-bacterial, immune-stimulating, and anti-viral effects (1-2). So far, baicalin has been demonstrated to have a wide range of anti-tumor activities *in-vitro* and *in-vivo*, while has insignificant effects on human normal hematopoietic cells and tissue cells (3). Based on the reports from earlier investigators, it was indicated that baicalin could induce the apoptosis of human colon cancer (4), breast cancer (5), lung carcinoma (6) and Burkitt lymphoma (7). Recently, it was reported that baicalin seemed to be very attractive as a new anticancer drug and a potential chemotherapeutic agent against human high-grade mucoepidermoid carcinoma (8). However, the effects and exact mechanism of baicalin on cervical cancer are still not known.

Cervical cancer is one of the most common gynecological malignancies, and its incidence is ranked the second in gynecological tumors. In recent years, the incidence of cervical carcinoma in young women under 35 years old is keeping rising with the increase of HPV infection (9). Currently, surgery, radiotherapy, chemotherapy and combined therapy are used to treat cervical cancer, however, these treatments could easily lead to adverse reactions or serious complications. Previous studies suggested that baicalin could inhibit the growth of a wide range of cancer cells, but had few side effects on normal cells, thus it could be a better selectivity for the treatment of human cancer. Therefore, exploring the effects and mechanism of baicalin on the apoptosis of cervical cancer HeLa cells has great theoretical and practical significance, which would serve to provide a new effective prevention and treatment methods for the treatment of cervical cancer.

## Experimental

*Chemicals and reagents*

Baicalin was provided by Nanjing ZeLang Medical Technology Co., Ltd. in China, and the determined purity was 98.12%, with Lot number ZL101005. DMEM/HIGH GLUCOSE was obtained from Thermo Fisher Scientific Inc. Domestic superior grade fetal bovine serum and Annexin V-FITC apoptosis detection kit were purchased from Nanjing KeyGen Biotech.Co., Ltd. in China. MTT cell proliferation and cytotoxicity assay Kit, Caspase-3 activity assay Kit, BCA protein assay kit, Dimethyl sulfoxide(DMSO), Trypsin-EDTA solution, Protein molecular weight marker, Cell lysis buffer for Western and IP, HRP-labeled goat anti-rabbit IgG (H+L) and PVDF membrane were purchased from Beyotime Institute of Biotechnology in China, while rabbit anti-Bcl-2, anti-Bax, anti-Fas, anti-FasL, anti-Caspase-8 and *β*-actin were obtained from Beijing biosynthesis biotechnology Co., Ltd. in China. All other chemicals and reagents used were of analytical grade.

*Cell culture*

HeLa cells obtained from Tumor Hospital of the Chinese Academy of Medical Sciences were cultured in DMEM containing 10% fetal bovine serum(FBS), 100 u/mL penicillin and 100 u/mL streptomycin in a humidified incubator at 37 °C under a 5% CO_2_ atmosphere. Cells were passaged every two to three days.

*Drug preparation*

Baicalin was dissolved in 100% DMSO at a concentration of 60 mg/mL as a stock solution and stored at 4 °C, it was diluted with DMEM medium before each experiment. The final concentrations of DMSO were less than 0.1% in all baicalin groups.

*MTT assay*

The effects of baicalin on HeLa cells viability were determined by a colorimetric MTT assay. HeLa cells were cultured in DMEM until the mid exponential phase, and then seeded in a 96-well plate at a density of 5×10^4^/mL per well in 100 μL of medium. After incubation for 24 h, the cells were exposed to baicalin (25, 50, 75, 100, 150, 200 μg/mL) or 10 μL DMEM (control) for 24 h under an atmosphere of 5% CO_2_ and 37 °C. After treatment, 10 μL of 5 mg/mL MTT was added, and the cells were incubated for 4 h at 37 °C. Then 100 μL Formanzan was added to dissolve the formazan crystals for 4 h, and the spectrophotometric absorbance at 570 nm was measured using a microplate reader .The inhibition rate was calculated as follows:

Inhibition rate (IR) (%) = (1–OD_treated_/OD_control_)×100%

IC_50_ value was calculated by regression curve of IR at different concentrations.

*Cell scratch* *method*

HeLa cells were seeded into six-well plates; straight lines were drawn by a pipette tip on the bottom of culture plate when cells spread to 80%-90% of the culture plate. Cells were washed with PBS to remove detached cells. Then cells were separately cultured in complete medium containing high dose (100 μg/mL) and low dose (50 μg/mL) baicalin, whereas cells of control group were incubated in complete medium. In the same multiple, cell migration into the scratch surface of Mark Points was observed by microscopy at 0-, 12-, and 24-h time points respectively. Edge distances of scratches were measured in photos and analyzed by computer software. The migration distance ratio was calculated as follows:

Migration distance ratio (%) = (1 – scratch distances at 24-h time point / scratch distances at 0-h time point)×100%

*Cell morphological assessment* *by* *inverted microscopy*

HeLa cells in the logarithmic phase were seeded in a 6-well plate. After incubation for 24 h, high dose (100 μg/mL) and low-dose (50 μg/mL) of baicalin were added into treated group respectively, while the same volume of DMEM medium was added into control group. After treatment for 24 h and 48 h, cell morphology was viewed by inverted microscopy. 

*Cell morphological observation* *by* *TEM*

HeLa cells were cultured for 24 h, high dose (100 μg/mL) and low-dose (50 μg/mL) of baicalin, and the same volume of DMEM medium were separately added into treated group and control group, cultured continually at 37 ^o^C under a 5% CO_2_ atmosphere. After treatment of baicalin for 48 h, cells were collected and fixed in 2.5% glutaraldehyde for 12 h at 4 °C, then fixed in 1% osmium tetroxide, dehydrated with graded concentrations of acetone and embedded in epoxy resin. Ultrathin sections were cut and stained with uranyl acetate for 30 min and then with lead citrate for 20 min, finally ultrastructure of apoptotic cells was examined by TEM (transmission electron microscope).

*Annexin V-FITC/PI flow cytometric analysis*

HeLa cells were treated with high and low dose baicalin for 48 h, then harvested and washed twice with PBS. 300 mesh strainers for filtration was used to obtain single cell suspension. 1×10^6 ^cells were counted and resuspended in 500 μL binding buffer, then incubated with 5 μL Annexin V-FITC and 5 μL PI for 10 min in the dark. HeLa cells were assayed by FACSCalibur flow cytometer, and cell apoptotic rate was obtained with ModFIT software. 

*Western blot* *analysis*

The expression of apoptosis-associated genes was investigated by Western blot analysis. HeLa cells(1×10^6^/mL) were seeded in a plate, then cultured for 24 h. After treatment with baicalin for 48 h, HeLa cells were collected and washed twice with cold PBS. The cells were then lysed in lysis buffer for Western and IP. Protein concentrations were quantified using BCA protein detection assay kits. Briefly, equal amounts of proteins were separated by SDSPAGE and transferred to a PVDF membrane. After blocking with 5% nonfat skim milk, the membrane was incubated overnight with the primary antibodies, and then with the secondary antibodies. Anti-rabbit Bcl-2, Bax, Fas, FasL, Caspase-8 and β-actin antibodies were used as the primary antibodies with horseradish peroxidase (HRP)-conjugated goat anti-rabbit IgG as secondary antibodies. After washing, proteins were detected with Western blot chemiluminescence assay, and the images were obtained by the TMW type gel imaging system. 

*Caspase-3 activity assay*

The activation of caspase-3 was detected by spectrophotometric method. 5×10^6^ cells were collected from the HeLa cells treated with high and low dose of baicalin for 12 h. Then cold lysis buffer was added into cells, and cells were lysed on ice, oscillated in vortex oscillator and centrifuged at 10,000 rpm for 1 min. After that the supernatant was drew to another centrifuge tube, protein concentrations were determined by bicinchoninic acid (BCA) method. 50 μL 2×reaction buffer and 5 μL Caspase-3 substrate were added into 50 μL cell lysis supernatant containing 200 μg protein, while 50 μL lysis buffer and 50 μL 2×reaction buffer were added into the control group. After culture at 37 °C for 4 h in dark, the absorbance at 450 nm was measured using a microplate reader, and caspase-3 activity was represented by the value of OD_treated_/OD_control_. 

*Statistical analysis*

Statistical analyses were performed using SPSS13.0 statistical software. Data were analyzed by one-way analysis of variance; differences between groups were tested by unpaired Student’s t-tests and expressed as the mean ± SD. A difference at: *p* <0.05 was considered to be statistically significant.

## Results

*Effect of baicalin on the growth of HeLa cells*

**Figure 1:**
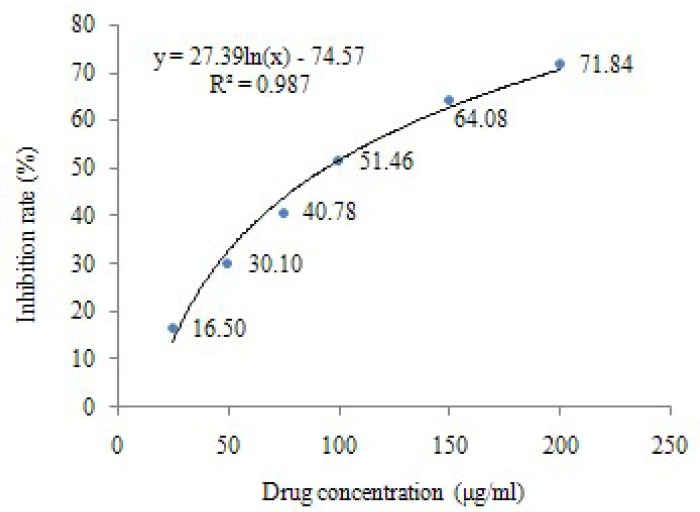
Effect of baicalin on the growth of HeLa cells.

The effect of baicalin on HeLa cells viability was determined by MTT assay. After the treatments with different concentrations of baicalin for 24 h, the results showed that the growth of HeLa cells was significantly inhibited, and the number of viable cells decreased in a dose-dependent manner (*P*<0.05, *P*<0.01). The inhibition rates of HeLa cells were shown in Table1, and the IC_50_ of baicalin (24 h) calculated by regression curve was 94.44 μg/mL (Figure 1).

**Table 1:** Effect of baicalin on the growth of HeLa cells.

**Group**	**OD**	**Inhibition rate (%)**
Control	1.03±0.09	—
Baicalin (25 μg/mL)	0.86±0.06*	16.50
Baicalin (50 μg/mL)	0.72±0.05*	30.10
Baicalin (75 μg/mL) Baicalin (100 μg/mL) Baicalin (150 μg/mL) Baicalin (200 μg/mL)	0.61±0.05** 0.50±0.04* 0.37±0.02** 0.29±0.02**	40.78 51.46 64.08 71.84

n = 10; (mean ± S.D., %);

* *P*<0.05,

** *P*<0.01 as compared with control group.

*Effect* *of* *baicalin on the proliferation and migration ability of HeLa cells*

**Figure 2:**
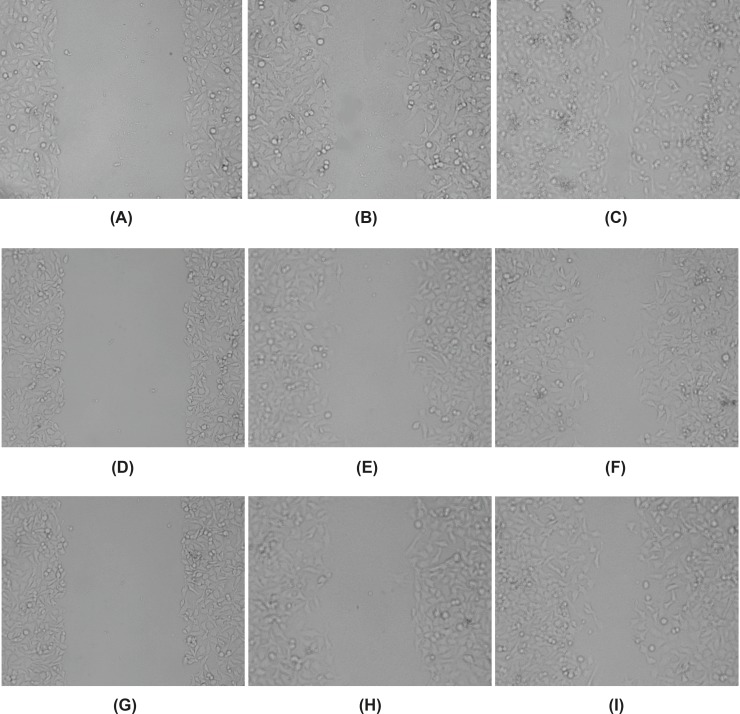
Effect on the proliferation and migration ability of HeLa cells treated with baicalin(×100). (A) control group(0 h),(B) control group(12 h), (C) control group(24 h), (D) high dose baicalin group(0 h), (E) high dose baicalin group(12 h), (F) high dose baicalin group(24 h), (G) low dose baicalin group(0 h), (H) low dose baicalin group(12 h), (I) low dose baicalin group(24 h).

Abilities of HeLa cells proliferation and migration were observed by cell scratch method. After scratching, cells in each group moved to the scratch area, but the speed of cells proliferation and migration was the fastest in control group, the scratch area almost disappeared at 24 h time point. The distance of cells migration in baicalin groups (50 μg/mL and 100 μg/mL) was significantly reduced, and the number of cells across the scratch area also decreased, especially the high dose group (100 μg/mL), the scratch area was still obvious at 24 h time point (Figure 2). Compared with the control group, the migration distance of baicalin groups decreased significantly (*P*<0.05), especially the cell migration ratio of high dose group was lower than that of low dose group (Table 2).The results showed that baicalin could inhibit the proliferation and migration of HeLa cells in a dose-dependent manner.

**Table 2:** Effect on the proliferation and migration ability of HeLa cells treated with baicalin.

**Group**	**Treatment (μg/mL)**	**Scratch** ** distance** ** (0 h)**	**Scratch** ** distance (24 h)**	**Migration distance ratio (%)**
Control	—	6.42±0.35	1.13±0.08	82.40±2.21
High dose of baicalin	100	6.44±0.29	3.28±0.21	49.07±2.38*
Low dose of baicalin	50	5.67±0.42	2.15±0.18	62.08±3.82*

n = 10; (mean ± S.D., %);

* *P*<0.05 as compared with control group.

*Morpholo* *gy of* *apoptotic HeLa cells induced by baicalin observed under light microscope*

**Figure 3:**
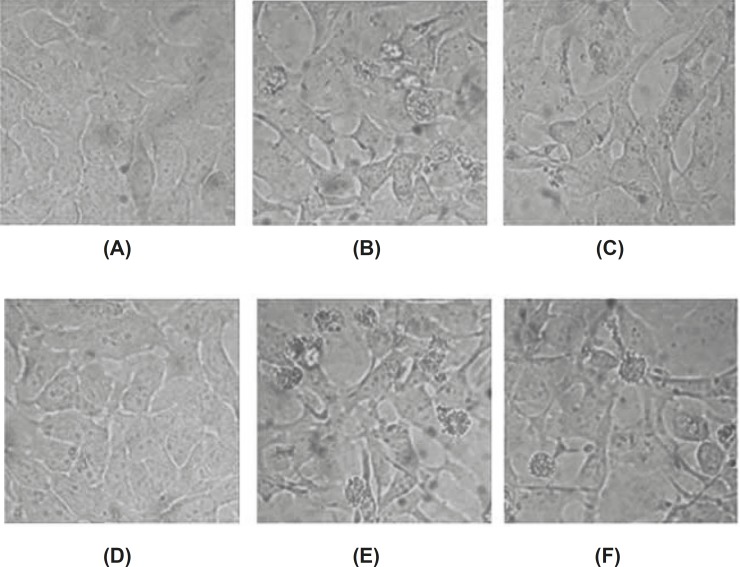
Morphological changes of apoptotic HeLa cells induced by baicalin observed under inverted microscopy (×400). (A) control group(24 h), (B) high dose group(24 h), (C) low dose group(24 h), (D) control group(48 h), (E) high dose group(48 h), (F) low dose group(48 h).

The conspicuous changes in the morphology of baicalin treated-cells were observed under inverted microscopy. HeLa cells grew well in the control group, whose shape was polygonal, color was even, and chromatin was loose. After the treatment with baicalin for 24 h and 48 h, to a certain extent, the growth of adherent cell was inhibited. Some cells became shrank and round, then cells detached from culture dish and floated. Moreover, part of cells became darker in color, cell membrane protruded, and apoptotic body appeared finally. These morphology changes of apoptotic cells demonstrated that baicalin had the ability to induce the apoptosis of HeLa cells. (Figure 3)

*Morpholog* *y* *of apoptotic HeLa cells induced by baicalin observed by TEM*

**Figure 4:**
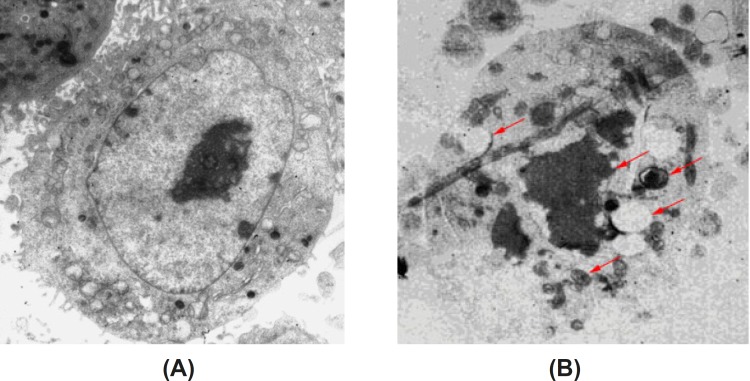
Morphology of apoptotic HeLa cells induced by baicalin observed by TEM(×6000). (A) control group, (B) high dose baicalin group.

Under the transmission electron microscope, we observed that there were rich microvilli on the surface of cells in the control group. Some organelles, such as mitochondria and rough endoplasmic reticulum, can be observed easily. Moreover, the cells possessed round nuclei, loose chromatin and large prominent nucleolus. HeLa cells were induced by high dose of baicalin for 48 h, the surface microvilli were significantly reduced, and meanwhile, chromatin condensation and the crescent-shaped body could be observed. These changes are the characteristic of apoptotic cell death (Figure 4).

*Detection of the percentage of apoptotic cells by a double staining method with Annexin V-FITC / PI*

**Figure 5:**
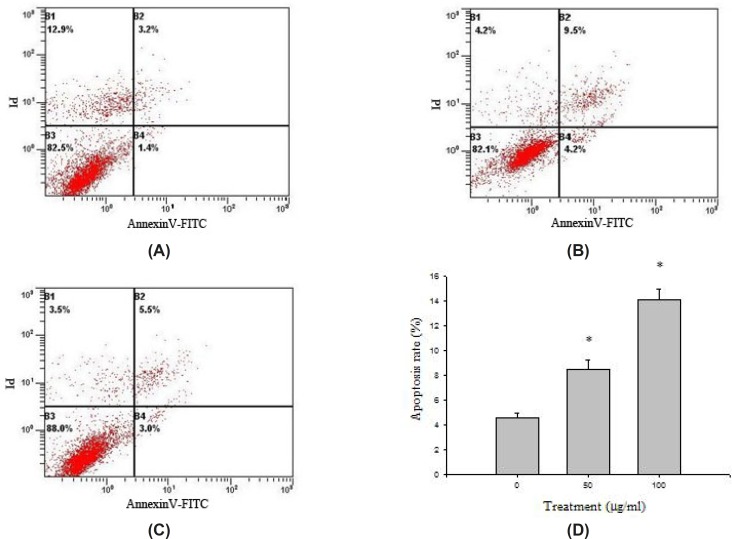
Detection of the percentage of apoptotic cells by flow cytometry. (A) control group, (B) high dose group, (C) low dose group, (D) effect of baicalin on HeLa cells apoptosis. ^*^P<0.05 as compared with control group

HeLa cells collected in all groups were stained through AnnexinV-FITC and PI, and then the cell apoptosis rates were detected by flow cytometry (Figure 5). HeLa cells were distributed into four quadrants: viable cells (Annexin−/PI−), early apoptotic cells (Annexin+/PI−), late apoptotic cells (Annexin+/PI+), and necrotic cells (Annexin−/PI+). The cell apoptosis rate in control group (AnnexinV - FITC positive, B2 + B4) was 4.61 ± 0.34%. Compared with the control group, the apoptosis rate of HeLa cell treated with baicalin was increased (*P*<0.05). The apoptosis rate of high dose group was more than 10%, higher than that of low dose group, which indicated that baicalin could significantly induce the apoptosis of HeLa cells in a dose-dependent manner. 

*Protein expression of apoptosis-related genes examined by Western blot*

*Effect of baicalin on Bcl-2 and Bax protein expression*

**Figure 6:**
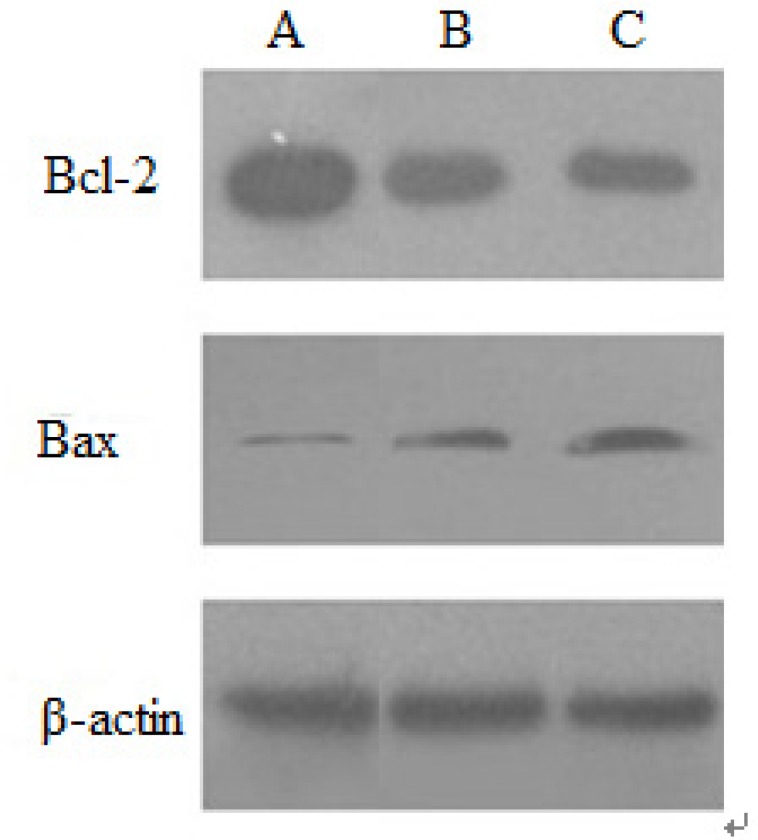
Expression of Bcl-2 and Bax protein induced by baicalin in HeLa cells.(A) control group, (B) low dose group, (C) high dose group

The protein expression of apoptosis-related genes induced by baicalin was examined by Western blotting. In no-treated HeLa cells, Bcl-2 protein strips can be described as: the color was darker, the areas were larger, the expression of protein was more. Moreover, in HeLa cells treated by baicalin for 48 h, the color of Bcl-2 protein became lighter, which showed that baicalin down-regulated the expression of Bcl-2 protein. The expression of Bax protein increased with the increasing of baicalin dose, indicating that baicalin could up-regulate Bax protein expression. The expression level of β-actin protein was substantially independent of baicalin, so β-actin protein was regarded as the internal reference. These results demonstrated that the expression of anti-apoptotic protein Bcl-2 was inhibited by baicalin in a dose-dependent manner, whereas that of Bax was inducted (Figure 6).

*Effe* *ct of* *baicalin on Fas,* *FasL and Caspase-8 protein expression*

**Figure 7:**
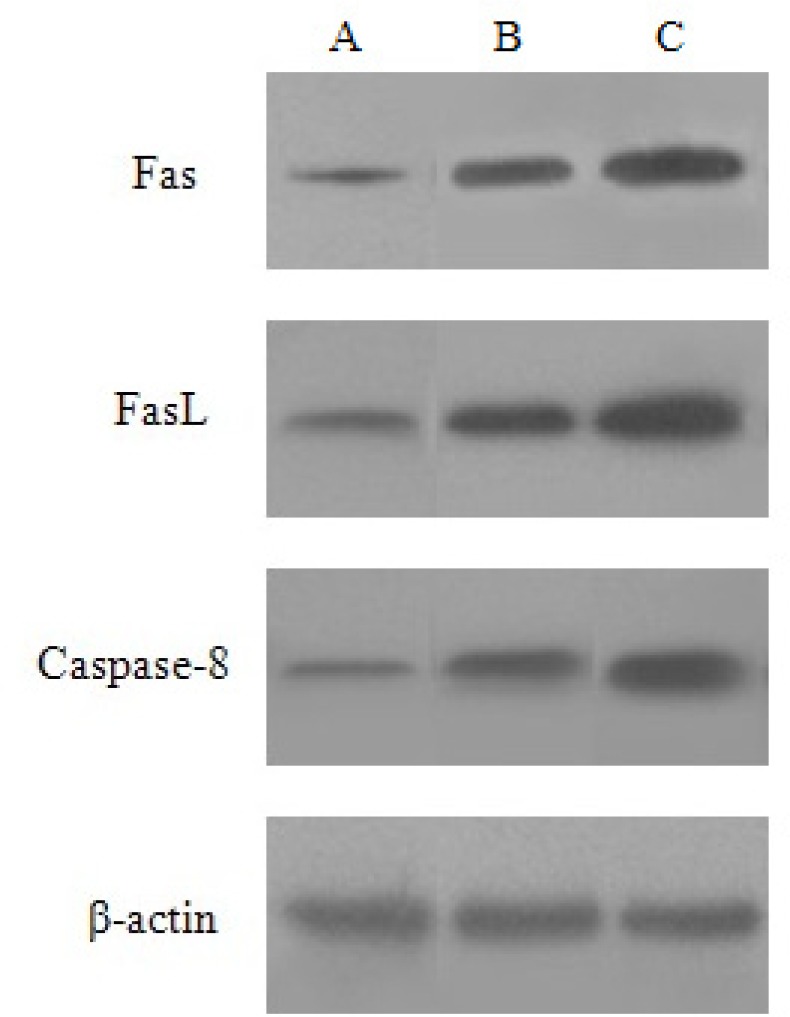
Expression of Fas, FasL and Caspase-8 protein induced by baicalin in HeLa cells. (A) control group, (B) low dose group, (C) high dose group.

HeLa cells were treated with the high and low dose of baicalin for 48 h. Compared with the control group, the expression of Fas, FasL and Caspase-8 protein increased, besides, the protein expression in high dose group was higher than that of the low dose group. *β-*actin was continually regarded as the internal reference (Figure 7). 

*The activation of caspase-3 investigated by spectrophotometric method*

After treatment with baicalin for 12 h, 24 h, 36 h and 48 h, the activation of Caspase-3 was detected by spectrophotometric method. As shown in Table 3, in baicalin-treated cells, Caspase-3 was gradually activated with the extension of time, while the activation of Caspase-3 in control group remained almost unchanged. Compared with the control group, Caspase-3 activation in baicalin group was increased unobviously after 12 h. But caspase-3 activation was significantly enhanced with the extension of treatment time after 24 h. Caspase-3 activation process in the high dose baicalin group was faster than that of low dose baicalin group, which revealed that baicalin induced the activation of caspases-3 in a time as well as dosed-dependent manner.

**Table 3:** Effect of baicalin on Caspase-3 activity of HeLa cells.

**Group**	**Treatment ** **(μg/mL)**	**Treatment time** **(h)**			
		**12**	**24**	**36**	**48**
Control	—	0.08±0.01	0.10±0.02	0.12±0.01	0.15±0.02
High dose of baicalin	100	0.17±0.02#	0.27±0.03*	0.39±0.03*	0.65±0.04**
Low dose of baicalin	50	0.12±0.01*	0.18±0.01*	0.31±0.02*	0.53±0.02**

n = 10; (mean ± S.D., %);

* *P*<0.05,

** *P*<0.01,

# *P*>0.05 as compared with control group.

## Discussion

As is well-known, apoptosis is a fundamental life phenomenon through the whole process of life. It has been reported that in many human tumor cells, the proliferation of cells will be unrestricted if cells apoptosis is severely hampered (10). So the balance between cell proliferation and apoptosis has a strong impact on the development and maintenance of normal organs (11). In further large scale studies, it has found that the most anti-cancer drugs in clinical application could induce tumor cell apoptosis. It was reported that baicalin exerted anti-tumor effects on human cancer cell lines through multiple apoptosis pathways (12-13): (i) the intracellular mitochondrial pathway (‘intrinsic’); (ii) the surface death receptor pathway (‘extrinsic’); (iii) the endoplasmic reticulum pathway. The induction of apoptosis in human cervical cancer by baicalin was reported, the research showed that baicalin hydrate could induce apoptosis via inducing mitochondrial dysfunction disrupted as shown as the mitochondrial membrane potential (14), while our results demonstrated that baicalin induced HeLa cells apoptosis through the activation of caspase-3 via both mitochondrial pathway and death receptor pathway. 

Our study observed the apoptosis of HeLa cells treated by baicalin for detecting the antineoplastic effects of baicalin on human cervical carcinoma. Cell viability was examined in an MTT assay. It was found that baicalin could significantly inhibite the growth of HeLa cells in time and dose dependent manner. In further research, the cell scratch assay was used to observe the abilities of HeLa cells proliferation and migration, the results showed that the speed of cell migration significantly decreased after treatment with baicalin, in contrast to the control group, the distance of cell migration was reduced as dose dependent.

Cell apoptosis is an active physiological process resulting in cellular self-destruction (15). Besides biochemical changes, it is characterized by distinct morphologic changes, including cell shrinkage, plasma membrane blebbing, chromatin condensation, increasing of cell density, karyorrhexis and the formation of apoptotic bodies. These changes can be observed by inverted biologic microscope and transmission electron microscope after HeLa cells treated by baiclain.

Furthermore, flow cytometry was performed to determine whether the baicalin-induced reduction in viability was attributable to apoptosis. The examination showed that the apoptosis rate of HeLa cells increased along with the increasing of baicalin concentration, moreover, the apoptosis rate of high dose group reached as high as 14.07 ± 0.85%. These results indicated that baicalin had the ability to induce apoptosis in HeLa cells.

The Bcl-2 family members may play a pivotal role in deciding whether a cell will live or die, because they reside upstream of irreversible cellular damage and focus much of their efforts at the level of mitochondria (16). Due to the apoptosis effect of the Bcl-2 family, it can be divided into pro-apoptotic protein (Bax, Bak, Bik, et al) and anti-apoptotic protein (Bcl-2, Bcl-xl, Bcl-w, et al). Especially, the Bax and Bcl-2 are two kinds of typical apoptosis protein. It has been reported that the ratio of pro-apoptotic Bax and anti-apoptotic Bcl-2 members is a critical determinant of susceptibility to apoptosis (17). The study by Zheng J showed that the protein expression levels of Bcl-2 decreased in HL-60/ADR cells after baicalin treatment while those of the pro-apoptotic gene gradually increased (18). Our results showed that baicalin can down-regulate Bcl-2 protein expression, and up-regulate Bax protein expression, as a result of which, the ratio of Bax/Bcl-2 increased significantly, inducing apoptosis of HeLa cells. And this is also consistent with Bhutia’s research(19). Moreover, baicalin also inducted the expression of Fas, FasL and Caspsase-8 protein in a dose dependent manner. Our results indicated that baicalin induced apoptosis in HeLa cells via Caspase-8 and Caspase-3 processing, in a Fas/FasL-dependent manner. Death receptor (Fas/FasL/Caspase-8) signal pathway is an important signal transduction system, which mediates cellular responses to the growth, differentiation and apoptosis of tutor cells. Death receptors belong to the tumour necrosis factor (TNF) receptor superfamily. Fas receptor–ligand interactions utilize caspase-8 activation to trigger down stream executioner caspase (20).

The central executioners of apoptosis, caspases, cleave numerous vital cellular proteins to affect the apoptotic cascade (21). In particular, Caspase-3 called the death protease，plays a key role during the process of apoptosis. The spectrophotometric assay was used to detect Caspase-3 activity. The results showed that in baicalin-treated cells, Caspase-3 activity increased, especially in high dose group, Caspase-3 activity was significantly inducted in a dose- and time-dependence. Our data clearly demonstrated that apoptosis rates of HeLa cells were associated with Caspase-3 activity, the percentage of apoptotic cells increased, while Caspase-3 activity increased apparently at the same time, and this is consistent with the research of Pidgeon (22).

It is well established that activation of a caspase cascade during apoptosis occurs via the activation of either the intracellular mitochondrial or the surface death receptor pathway (23). Therefore, it was deduced that baicalin-induced apoptosis involved the activation of Caspase-3 in HeLa cells via these two pathways. The present studies demonstrated that baicalin increased the ratio of Bax/ Bcl-2 to induce cell apoptosis by regulating Bcl-2 and Bax protein expression via the intracellular mitochondrial pathway. The increasing of Bax/Bcl-2 ratio will alter mitochondrial membrane permeability, result in the release of cytochrome C in mitochondrion, moreover, cytochrome C combines with other factors, leading to activate Caspase-3 and cell apoptosis (24). In this study, it was also shown that baicalin promoted the levels of Fas/FasL and activated caspases-8, which then activated the downstream signal (caspases-3) further through the cell surface death receptor pathway. 

Our study demonstrated that baicalin could inhibit the proliferation of HeLa cells via the mechanism involving the induction of apoptosis through the intracellular mitochondrial pathway and the surface death receptor pathway. So we believe that baicalin may be as a candidate in the development of anti-cancer drugs. 
